# Genetic Risk Scores Identify People at High Risk of Developing Diabetic Kidney Disease: A Systematic Review

**DOI:** 10.1210/clinem/dgad704

**Published:** 2023-12-01

**Authors:** Aleena Shujaat Ali, Cecilia Pham, Grant Morahan, Elif Ilhan Ekinci

**Affiliations:** Department of Medicine, The University of Melbourne, Melbourne 3084, Australia; Australian Centre for Accelerating Diabetes Innovations, The University of Melbourne, Melbourne 3000, Australia; Department of Medicine, The University of Melbourne, Melbourne 3084, Australia; Australian Centre for Accelerating Diabetes Innovations, The University of Melbourne, Melbourne 3000, Australia; Australian Centre for Accelerating Diabetes Innovations, The University of Melbourne, Melbourne 3000, Australia; Diabetes Research Foundation, The University of Western Australia, Perth 6009, Australia; Department of Medicine, The University of Melbourne, Melbourne 3084, Australia; Australian Centre for Accelerating Diabetes Innovations, The University of Melbourne, Melbourne 3000, Australia; Department of Endocrinology, Austin Health, Melbourne 3084, Australia

**Keywords:** diabetic kidney disease, genetic risk score, polygenic risk score

## Abstract

**Context:**

Diabetic kidney disease (DKD) is the leading cause of end-stage renal disease. Measures to prevent and treat DKD require better identification of patients most at risk. In this systematic review, we summarize the existing evidence of genetic risk scores (GRSs) and their utility for predicting DKD in people with type 1 or type 2 diabetes.

**Evidence Acquisition:**

We searched MEDLINE, Embase, Web of Science, and Cochrane Reviews in June 2022 to identify all existing and relevant literature. Main data items sought were study design, sample size, population, single nucleotide polymorphisms of interest, DKD-related outcomes, and relevant summary measures of result. The Critical Appraisal Skills Programme checklist was used to evaluate the methodological quality of studies.

**Evidence Synthesis:**

We identified 400 citations of which 15 are included in this review. Overall, 7 studies had positive results, 5 had mixed results, and 3 had negative results. Most studies with the strongest methodological quality (n = 9) reported statistically significant and favourable findings of a GRS’s association with at least 1 measure of DKD.

**Conclusion:**

This systematic review presents evidence of the utility of GRSs to identify people with diabetes that are at high risk of developing DKD. In practice, a robust GRS could be used at the first clinical encounter with a person living with diabetes in order to stratify their risk of complications. Further prospective research is needed.

A third of people with diabetes can develop diabetic kidney disease (DKD) (1, 2). It is the leading cause of end-stage renal disease (ESRD) (1, 3-6). Measures to prevent and treat DKD require better patient stratification.

Genome-wide association studies (GWAS) have discovered genetic variants associated with DKD risk, but translation of these findings into clinical benefit is difficult because individual variants explain a relatively small proportion of overall risk (7). A genetic risk score (GRS), however, aggregates the individual effects of variants to increase their predictive power (7). The aim of this systematic review was to summarize the existing evidence of using GRSs to identify people at high risk of developing DKD.

## Research Design and Methods

### Search Strategy and Selection Criteria

The protocol for this systematic review was registered on PROSPERO (ID: CRD42023402057). We searched MEDLINE, Embase, Web of Science, and Cochrane Reviews on the June 29, 2022, for all relevant articles. In consultation with a medical librarian, a search hedge was created in 4 parts: (1) terms related to “genetics,” (2) terms related to “diabetes” including “type 1 diabetes” and “type 2 diabetes,” (3) terms related to “kidney disease,” and (4) terms restricted to “score” or “signature.” In addition to database searches, we searched reference lists of relevant reviews. Following the removal of duplicate studies, 2 authors independently screened the titles, abstracts, and full texts and a third author confirmed inclusion of these studies. We identified studies in which a genetic risk score was used to identify the risk of DKD in people with type 1 or type 2 diabetes. All identified articles from the literature search were entered into Covidence for screening.

A study was excluded if it (1) did not statistically compare outcomes between groups; (2) included people with monogenic diabetes, gestational diabetes, glucocorticoid-induced diabetes, or pancreatic insufficiency; (3) investigated kidney disease secondary to conditions other than diabetes; (4) was not published in English; or (5) was not available as full text (ie, only an abstract was available).

### Data Analysis

After study selection, 1 author extracted data from the articles and stored them in Microsoft Excel. Main data items sought in this stage were study design, sample size, population, single nucleotide polymorphisms (SNPs) of interest, main DKD-related outcome or outcomes, and relevant summary measures of results (odds ratios, hazard ratios, *P-*values). A study was classified as having positive results if the GRS showed a statistically significant ability to predict all DKD-related outcomes. The study was classified as having mixed results if at least 1 but not all of the outcomes of interest was statistically significant. A study was classified as having negative results if none of the outcomes of interest showed statistical significance.

We used a checklist based on the Critical Appraisal Skills Programme checklist (8) to evaluate the methodological quality and risk of bias of studies included in our systematic review. The checklist consisted of 10 items across 3 domains: section A: validity (7 items), section B: results (3 items), and section C: relevance (2 items). Items were rated as either “yes” (1 point) or “no/unsure” (0 points). This resulted in a maximum total score of 12 points, with higher scores indicating a stronger methodological quality. On the basis of their total score across all domains, studies were classified into 1 of the following methodological quality categories: excellent (11-12 points), good (9-10 points), fair (7-8 points), poor (6 points or less).

## Results

**Figure 1.**
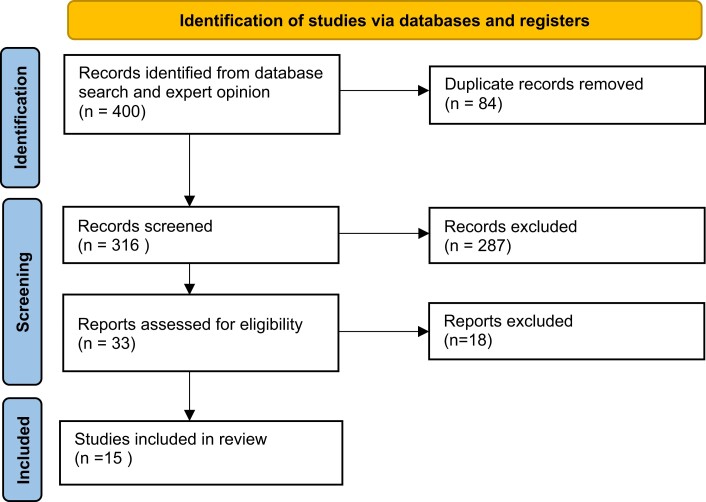
Study selection.

We identified 400 citations from 4 databases (Fig. 1). After the removal of duplicates (n = 84), 316 titles and abstracts were screened, 287 of which were excluded. Following full text review of 33 articles, 15 studies were included in this review on the basis of selection criteria (Table 1). Overall, 7 studies had positive results, 5 had mixed results, and 3 had negative results.

**Table 1.** Study characteristics

Outcome	Study	Study design	Sample size	Population	Diabetes type	SNPs of interest	Measurement of renal outcomes	Quality
**Positive**								
GRS associated with low eGFR, macroalbuminuria, microalbuminuria, major microvascular events, micro- and macro-vascular disease, new/worsening nephropathy	Tremblay 2021	Case control study	4098	European	T2D	598 SNPs associated with micro- and macrovascular outcomes in addition to their common risk factors	eGFR, macroalbuminuria, new or worsening nephropathy, major microvascular events	Excellent
GRS of severe insulin resistance and relative insulin insufficiency was associated with progressive CKD	Wang 2012	Cohort study	1386	South East Asians	T2D	35 SNPs associated with insulin secretion and 20 SNPs associated with insulin sensitivity	Progressive CKD	Excellent
GRS of higher BMI associated with DKD, macroalbuminuria and ESRD	Todd 2015	Case control study	5209	European	T1D	32 SNPs associated with obesity	DKD. macroalbuminuria, ESRD	Excellent
Risk of DKD increased with increasing GRS	Liao 2019	Case control study	1514	Chinese	T2D	7 SNPs associated with DKD in Han Chinese	DKD	Good
GRS associated with increased risk of CKD	Vujkovic 2020	Case control study	67 403	European	T2D	558 SNPs associated with vascular outcomes in T2D	CKD	Fair
GRS associated with increased risk of DKD/coronary artery disease; GRS associated with cumulative micro- and/or macrovascular complications	Rattanatham 2021	Case control study	1700	Thai	T2D	5 SNPs in the *TCF7L2* and *KCNQ1* genes	Cumulative DKD and/or coronary artery disease	Fair
Higher GRS associated with increased odds of rapid decline in kidney function; higher genetically predicted plasma uric acid associated with rapid decline in kidney function	Gurung 2022	Case control study	2485	Chinese	T2D	GRS for plasma uric acid	Rapid decline in kidney function	Fair
**Mixed**								
GRS of increased waist-hip ratio associated with ESRD, GRS of increase BMI associated with DKD	vanZuydam 2018	Case control study	10 873	UK	T1D + T2D	GRS of 20 traits related to diabetes, insulin resistance, obesity, hypertension, lipids, coronary artery disease. 10-96 SNPs per phenotype	ESRD, DKD	Excellent
GRS associated with decreased eGFR but not microalbuminuria	Xu 2016	Cross sectional study	11 502	Chinese	T2D	34 SNPs associated with susceptibility to T2D	eGFR, uACR	Good
GRS associated with lower eGFR, no association between GRS and albumin excretion rate	Zusi 2018	Cross sectional study	1591	Italian	T2D	39 SNPs related to risk of kidney disease and 42 SNPs related to cardiovascular risk	eGFR, albumin excretion rate	Good
GRS for T2D was associated with severe autoimmine diabetes, severe insulin deficient diabetes, mild obesity-related diabetes, mild age-related diabetes but not with severe insulin resistant diabetes (which is the cluster with highest incidence of CKD, macroalbuminuria, ESRD); GRS for insulin secretion was associated with mild obesity-related diabetes, mild age-related diabetes and severe insulin deficient diabetes; GRS for insulin resistance was not associated with any cluster	Ahlqvist 2018	Cluster analysis	3747	Swedish	T1D + T2D	5 SNPs for insulin resistance, 16 SNPs for insulin secretion, 65 SNPs for T2D	eGFR, CKD, ESRD, macroalbuminuria	Good
GRS for beta cell and proinsulin associated with severe insulin resistant diabetes (indicating higher beta cell function) but not GRS for obesity, liver, or lipodystrophy	Slieker 2021	Cross sectional study	12 828	Scandinavian	T2D	394 SNPs for diabetes-related risk	SIRD cluster	Good
**Negative**								
GRS for beta cell dysfunction and insulin resistance not significantly associated with severe insulin resistant diabetes cluster	Wang 2022	Cluster analysis	687	Singaporean	T2D	35 SNPs associated with insulin secretion; 20 SNPs associated with insulin sensitivity; 9 SNPs for T1D	Progressive CKD	Good
Risk of renal events did not differ according to GRS; GRS was not significantly related to eGFR trajectory	Barbieux 2019	Cohort study	1619	French	T1D + T2D	18 SNPs associated with renal function and CKD 5	Renal events (doubling of serum creatinine), ESRD requiring renal replacement therapy	Fair
GRS for diabetic retinopathy afforded no cumalitive effect on risk of DKD, eGFR status or ESRD outcomes	Hsieh 2020	Case control study	1476	Chinese	T2D	33 SNPs related to diabetic retinopathy	eGFR, ESRD	Poor

Abbreviations: BMI, body mass index; CKD, chronic kidney disease; DKD, diabetic kidney disease; ESRD, end-stage renal disease; eGFR, estimated glomerular filtration rate; GRS, genetic risk score; SNP, single nucleotide polymorphism; T2D, type 2 diabetes; T1D, type 1 diabetes; uACR, urinary albumin-creatinine ratio.

Results of the methodology quality assessments are reported in Supplementary Table 1 (9). All studies were observational in nature. Four studies (3 case-control studies and 1 cohort study) were categorized as excellent. Six studies (1 case-control study, 3 cross-sectional studies, and 2 cluster analyses) were categorized as good. Four studies (3 case-control studies and 1 cohort study) were categorized as fair. One study was categorized as poor, and this was a case-control study. The total quality design score ranged from 6 to 12 (poor to excellent), with a mean score of 9.4 (good). Within domains, mean scores were as follows: 5.9 points (out of 7) for validity, 2.3 points (out of 3) for results, and 1.3 (out of 2) for relevance. In general, studies that received a low score had differences in study groups that may have affected the outcome of interest, had a low odds ratio, or had low statistical significance.

Eleven studies investigated GRS in type 2 diabetes. Three studies investigated GRS in both type 1 diabetes and type 2 diabetes. One study investigated type 1 diabetes only. The number of SNPs that were used to construct the GRS ranged from 5 to 598 between studies. Most studies (9) investigated people with European ancestries and the rest (6) investigated Asian populations.

The single study investigating people with type 1 diabetes only (10) was a case-control study that investigated 32 SNPs associated with obesity in a European population. This study found an association with all 3 outcomes of DKD (broadly defined DKD, macroalbuminuria, and ESRD) with genetically determined elevated body mass index (BMI). Similarly, van Zuydam et al who investigated type 1 diabetes and type 2 diabetes in a UK cohort (11) constructed a weighted GRS for 20 traits related to diabetes, insulin resistance, obesity, hypertension, coronary artery disease, and lipids. These GRSs, constructed from signals identified in previously published GWAS, included between 10 and 96 SNPs per phenotype. A GRS for increased BMI was associated with all DKD phenotypes. Furthermore, a GRS for increased waist-to-hip ratio was associated with ESRD in subjects with type 2 diabetes in this cohort.

Three studies investigating GRS for DKD in type 2 diabetes used SNPs associated with chronic kidney disease (CKD) or DKD itself. Liao et al investigated 7 SNPs associated with DKD in Han Chinese people in previous GWAS (12). When added to a model of significant clinical predictors of DKD (age, obesity, abnormal triglycerides, hypertension, and heart disease), each additional risk allele in the GRS increased the risk of DKD by 1.24-fold [95% confidence interval (CI) 1.17-1.34]. In contrast, Barbieux et al constructed a GRS of 18 SNPs associated with renal function and CKD (5). In a cohort of French people with type 1 or type 2 diabetes, they found that risk of renal events did not differ according to GRS and that GRS did not significantly predict estimated glomerular filtration rate (eGFR) trajectory (13). Mixed results were seen by Zusi et al (14) who investigated a cohort of Italian people with type 2 diabetes. They constructed GRSs using 39 SNPs related to risk of kidney disease and 42 SNPs related to cardiovascular risk. After adjustment for a number of confounders, they found that the top GRS quintile had the lowest eGFR. No statistical significance, however, was detected between renal GRS and urinary album excretion rate.

Single nucleotide polymorphisms associated with other micro- and macrovascular diseases were also investigated in a further 3 studies. Vujkovic et al investigated a large cohort of European people with type 2 diabetes (15). They constructed a GRS from 558 SNPs associated with vascular outcomes in type 2 diabetes and a further 21 SNPs seen in European people only. The risk of DKD increased significantly with increased GRS. Similarly, Tremblay et al investigated a cohort of European people aged >65 years with type 2 diabetes (16). They identified 26 factors and outcomes that were grouped into 10 groups of risk/outcomes with 598 SNPs. Combined with sex, age at onset of diabetes, and diabetes duration, the GRS model allowed prediction of both microvascular and macrovascular endpoints of type 2 diabetes. Hsieh et al investigated whether SNPs for diabetic retinopathy had pleiotropic effects on DKD in Han Chinese people in Taiwan with type 2 diabetes and retinopathy (17). Their results suggested that the 33 SNPs that were investigated exerted no cumulative effect on the eGFR status, DKD risk, or ESRD risk among people with type 2 diabetes.

Four studies investigated SNPs related to insulin secretion, sensitivity, and/or resistance (11, 18-20). Wang et al examined the independent and joint effects of 35 SNPs associated with insulin secretion and 20 SNPs associated with insulin sensitivity on CKD in a prospective cohort of Chinese people with type 2 diabetes (20). After controlling for baseline confounding variables, each additional unit of a weighted GRS was associated with a 37.2% (*P* = 5.23 ×10^7^) increase in CKD risk. Compared to participants carrying 0 to 2 risk alleles or in the lowest quartile of the GRS, the adjusted risk for CKD was 3.2 (95% CI 1.97-5.07, *P* = 2.03 ×10^6^) and 2.5 (95% CI 1.56-3.85, *P* = 1.03 × 10^4^) carrying 5 or more risk alleles or in the highest quartile of the GRS.

Three of the studies investigating insulin secretion, sensitivity, and/or resistance were cluster analyses (18, 19, 21). Ahlqvist et al examined a large cohort of Swedish people with type 1 and type 2 diabetes. They classified people into subgroups of diabetes based on 5 variables: BMI, age of onset of diabetes, presence of glutamic acid decarboxylase antibodies, homoeostasis model assessment (HOMA) 2 estimates of β-cell function (HOMA2-B), and insulin resistance (HOMA2-IR) based on C-peptide concentrations. They described 5 “clusters” using these variables: severe autoimmune diabetes, severe insulin-deficient diabetes, severe insulin-resistance diabetes (SIRD), mild obesity-related diabetes (MOD), and mild age-related diabetes. Their analyses showed that SIRD was the cluster with the highest incidence of CKD, macroalbuminuria, and ESRD. They constructed GRS from 65 SNPs associated with type 2 diabetes and found this was associated with all clusters (*P* < .0008) except SIRD (*P* = .16). An insulin secretion risk score constructed from 16 SNPs was significantly associated with MOD (*P* = .0002) and mild age-related diabetes (*P* < .0001) but again showed no evidence of association with SIRD (*P* = .65).

Similarly, Wang et al subtyped Southeast Asian people with type 2 diabetes by de novo cluster analysis (19). They identified 3 novel subgroups of diabetes: MOD, mild age-related diabetes with insulin insufficiency (MARD-II), and severe insulin-resistant diabetes with relative insulin insufficiency (SIRD-RII). Over a median of 7.3 years’ follow-up, the SIRD-RII subgroup had the highest risks for progressive kidney disease, while the MARD-II subgroup had moderately elevated risk for kidney progression. They created a GRS for beta cell dysfunction and insulin resistance based on 35 SNPs associated with insulin secretion and 20 SNPs associated with insulin sensitivity, respectively, in Asian populations. A GRS for type 1 diabetes was constructed using 9 SNPs. They found that, compared to the MOD subgroup, the participants in the MARD-II subgroup had a significantly higher GRS for beta cell dysfunction. There was no significant difference in the GRS for beta cell dysfunction between the SIRD-II and MOD subgroups and no significant difference in the GRS for insulin resistance among the 3 subgroups.

A third study investigated the aetiology of the clusters escrybed by Ahlqvist by comparing their molecular signatures (21). This was a cross sectional study of more than 12 828 Scandinavian people with type 2 diabetes. They created a GRS from 394 SNPs related to diabetes-related risks and found that a GRS for beta cell and proinsulin was associated with the SIRD cluster [β-cell, β 1.41 (95% CI −2.21 to −.62); proinsulin, −.28 (95% CI [−.41 to −.15)]. In MOD, the GRS for obesity was significantly higher compared with the other clusters [β .51 (95% CI .34–.68)].

Xu et al investigated a large cohort of Chinese people with type 2 diabetes (22). They created a GRS by using 34 SNPs associated with susceptibility to type 2 diabetes that were previously identified in East Asians. They found that every SD increase in the GRS was associated with a 12% increased risk of eGFR (95% CI 1.04-1.20, *P* = .001). Compared with the lowest quartile of the GRS, the second, third, and highest quartiles were associated with 15%, 19%, and 34% increased risk of reduced eGFR, respectively (*P* for trend = .005). Positive results, however, were not found between GRS and increased urine albumin creatinine ratio.

Rattanatham et al conducted a case-control study to investigate the effects of combined gene polymorphisms within TCF7L2, KCNQ1, and KCNJ11 on vascular complications in Thai subjects with type 2 diabetes (23). Among the people with type 2 diabetes, there were no associations for any of the 5 individual SNPs with the complications. They found, however, that a combination of 2 risk alleles in KCNQ1 [rs2237892 (C) and rs2237897 (T)] revealed significant associations. The high-GRS group was associated with increased risks of cumulative nephropathy and/or coronary artery disease [odds ratio (OR), 3.49; 95% CI 1.49-8.15, *P* = .004]. There was a borderline association with cumulative micro- and macrovascular complications (OR, 2.06; 95% CI .97-4.39) as compared with a group with low GRS. A combination of multiple risk alleles was subsequently investigated, from which a significant association was found only for the combination of TCF7L2 rs7903146 (C), KCNQ1 rs2237892 (C), and KCNQ1 rs223797 (T). Compared with a group with a lower GRS, the high-GRS group revealed significant association with cumulative nephropathy and/or coronary artery disease (OR, 3.92; 95% CI, 1.75 to 8.76; *P* = .001), and cumulative micro- and/or macrovascular complications (OR, 2.33; 95% CI, 1.13 to 4.79; *P* = .022).

Finally, Gurung et al studied 2 cohorts of Chinese people to determine the association of a GRS for plasma uric acid and rapid decline in kidney function in people with type 2 diabetes (24). There was no statistically significant association in each individual cohort but when analyzing the cohorts together, the authors found that a higher GRS was associated with increased odds of rapid decline in kidney function (meta-adjusted OR 1.12; 95%CI 1.01-1.24, *P* = .030).

There were 17 SNPs that were recurrent throughout the studies with positive outcomes (Table 2). These were *KCNQ1* rs2237892, *CADM2* rs13078807, *ETV5* rs9816226, *TCF7L2* rs7903146, *MTCH2* rs3817334, *NRXN3* rs10150332, *MAP2K5* rs2241423, *SH2B1* rs7359397*, SLC39A8* rs13107325, *APOB* rs1367117, *TNNI3K* rs1514175*, NUDT3* rs206936*, BDNF* rs10767664*, FTO* rs1558902, *MTIF3* rs4771122, *TFAP2B* rs987237, and *TBL2* rs17145738. The gene whose variants were most commonly used to construct a GRS was *KCNQ1*. Tremblay et al used 3 variants in *KCNQ1,* Rattanatham et al used 2, and Gurung et al used 1. Each of the SNPs used in Liao et al's study was unique and did not recur in any of the other positive studies.

**Table 2.** SNPs that were seen to recur in the studies with a positive outcome

*Gene*	Tremblay 2018	Wang 2012	Todd 2015	Liao 2019	Vujkovic 2020	Rattanatham 2021	Gurung 2022
*LRP2*	rs4667594						rs2390793
*KCNQ1*	rs231362					rs2237897	
	rs2237892					rs2237892	
	rs163160						
*CADM2*	rs2325036		rs13078807				
	rs13078807						
*ETV5*	rs10513801		rs9816226				
	rs1516725						
	rs9816226						
*TCF7L2*	rs7903146					rs7903146	
*MTCH2*	rs3817334		rs3817334				
*NRXN3*	rs10150332		rs10150332				
	rs7144011						
*MAP2K5*	rs2241423		rs2241423				
	rs4776970						
*SH2B1*	rs7498665		rs7359397				
	rs7359397						
*UMOD*	rs4293393				rs12917707		
	rs13329952				rs3485707		
*LPL*	rs12678919	rs328					
	rs264						
	rs2083636						
*TENM3*	rs7692395				rs2177223		
*TGFB1*	rs8108632	rs1800469					
*KCNJ11*	rs5215					rs5219	
*SLC39A8*	rs13107325		rs13107325				
*APOB*	rs1367117	rs1367117					
*TNNI3K*	rs1514175		rs1514175				
*NUDT3*	rs206936		rs206936				
*BDNF*	rs10767658		rs10767664				
	rs10767664						
*FTO*	rs1558902		rs1558902				
	rs9939609						
*FGB*	rs1800789	rs1800790					
*MTIF3*	rs4771122		rs4771122				
*TFAP2B*	rs987237		rs987237				
*TBL2*	rs17145738						rs17145738
*BCAS3*	rs7212798						rs9895661
	rs8068952						
	rs1167044						

## Conclusions

In this systematic review, we identified 15 studies of genetic risk scores investigated worldwide between 2001 and 2022. The GRSs were designed to identify people at a genetically high risk of developing DKD, with the vast majority analyzing people with type 2 diabetes. Most studies with the strongest methodological quality assessment (n = 9) reported statistically significant and favorable findings of a GRS’s association with at least 1 measure of DKD.

**Figure 2.**
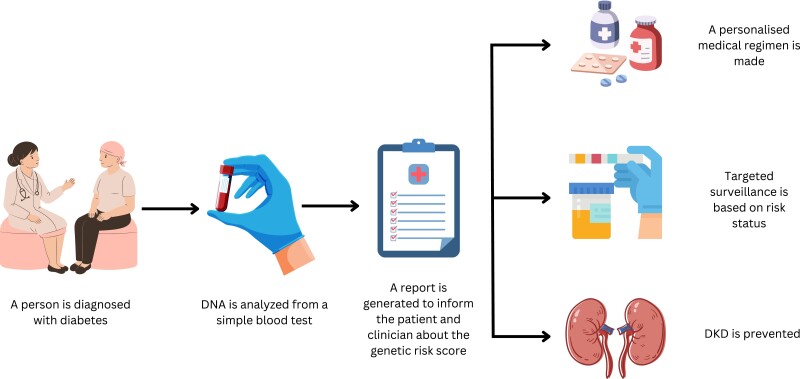
Flow chart showing how a genetic risk score can be integrated into clinical practice.

This systematic review is the first to support the utility of a GRS to identify people with diabetes that are at high risk of developing DKD. In practice, a robust GRS could be used at the first clinical encounter with a person living with diabetes (Fig. 2). A report could be generated from a routine blood test, allowing the clinician a targeted approach to diabetes medication selection and frequency of surveillance. If this is done at the time of diagnosis, this would be particularly useful and allow high-risk people to be prioritized for review earlier in the course of the disease. Renoprotective agents could then be used with close specialist surveillance in order to reduce DKD progression (25-27). This could ultimately prevent or delay the development of DKD.

From the perspective of genetic architecture, CKD does not represent a single disease but rather a highly heterogenous group of pathophysiological processes. This is reflected in the studies that we reviewed showing the association of DKD with SNPs related to obesity, vascular disease, metabolic syndrome, uric acid, and beta cell dysfunction.

Our systematic review included a significant proportion of studies conducted with Asian participants (7 out of 15 studies) (12, 17, 19, 20, 22-24). In view of the high prevalence of diabetes in these populations, it is encouraging to see an interest in genetic studies that target the early identification and prevention of DKD in people of Asian descent.

Our systematic review has a number of strengths. First, we examined GRSs for DKD using different combinations of SNPs and in many different populations. Second, we used a robust search strategy in consultation with a medical librarian and extracted articles from 4 databases.

This systematic review has several limitations. First, our search was restricted to articles in English, so we might not have fully characterized global efforts at identifying GRSs for DKD. Publication bias also means that our study favors our hypothesis. Second, heterogeneity in sample size, selection of SNPs, population, diabetes type, study design, and stage of DKD existed between all studies. Substantial variability in study characteristics made comparisons between studies difficult. For example, studies that measured more than 1 outcome had a greater chance of being classified as mixed.

Furthermore, the studies varied in their definition of DKD and measurement of renal endpoints. The Kidney Disease Improving Global Outcomes defines DKD as the presence of markers of kidney damage and GFR <60 mL/min/1.73 m^2^ for > 3 months (5). Several studies used progression of DKD (13, 19, 20, 24) or ESRD (10, 11, 13, 17, 18) as endpoints, which made direct comparisons between studies complex. Moreover, the studies did not rely on biopsy-proven diagnoses of DKD. Studies that describe biopsy-proven DKD are rare, and none could qualify for this review. Although this would increase accuracy for future GRS studies, in clinical practice biopsies are only performed when the aetiology of chronic kidney disease is unclear. Hypertension and IgA nephropathy can commonly coexist with long-standing diabetes and may confound the aetiology of DKD. This may be particularly important in certain populations like parts of China where glomerulonephritis remains the leading cause of chronic kidney disease (28). This must be taken into consideration, especially given that 7 out of the 15 studies included in this review were in Asian populations. Future GRS studies can consider renal biopsy for accurate diagnosis or exclusion of other common causes with renal ultrasound or urinalysis.

Our assessment of methodological quality highlights the opportunity for improvement in study rigor. Most studies (n = 5) were rated as good. More methodologically rigorous studies will enable firmer recommendations about the most effective GRS to identify DKD. Improvements could be made in all domains of study quality, including strengthening external validity (eg, demonstrating that a study population is representative of the source population) and ensuring that studies are adequately powered. Furthermore, the Critical Appraisal Skills Programme checklist that was used to assess methodological quality is inherently subjective. We tried to improve objectivity by integrating a score as a quantitative measure into this evaluation. It should be noted, however, that a degree of subjectivity of those reviewing the quality of the studies remains.

Our review is also limited by the number of studies available for analysis. This may be due to the relatively recent technical advancements in genetics that allows quicker and cheaper genetic typing and association studies of large cohorts of people. Furthermore, although it is relatively well understood that there is a genetic basis for the development and progression of DKD, the utility of GRSs remain elusive. The limited number of studies make interpretation of our findings less robust but also highlight the importance of further research in this area.

Research into GRSs has been fruitful and has led to the development of in vitro diagnostic tests and improved clinical practice. For instance, women in the United Kingdom are invited to start mammographic screening when they turn 47 years old, which corresponds to a 2.4% 10-year risk threshold, as this is the average risk for women at this age. According to a polygenic risk score using 77 SNPs, women in the top 10% of genetic risk reach this risk threshold in their early 30s , whereas women in the bottom 10% of the polygenic risk remain below this threshold throughout their lifetime (29, 30). Thus, information on genetic risk is more effective than an age-based criteria in guiding initiation of mammographic screening. In cardiovascular disease, a genetic risk score can identify individuals with a 4-fold increased risk for coronary artery disease. This risk is similar to monogenic conditions like familial hypercholesterolaemia (31). If a similar genetic risk score can be used to identify high and low risks for DKD, we could target screening and treatment options to those that need it most.

Consideration must also be given to the selection of SNPs for the most robust GRS for identification of DKD risk. For instance, certain studies explored an expansive set of genes (up to 598 SNPs in Tremblay et al's study), while other studies focused on only a small and specific number of genes. Our review highlighted 17 SNPs that were recurrent throughout studies that showed a positive outcome. The gene most often included in the scores was *KCNQ1,* which encodes the pore-forming α subunit of voltage-gated potassium channels (32) and was initially identified as a type 2 diabetes susceptibility gene (33). Further research then showed that it may also confer susceptibility to diabetic nephropathy, as evidenced by the presence of macroalbuminuria (34, 35). Whether variants in this gene would serve to enhance other GRSs for DKD requires further research.

The most recent literature suggests there is moderate evidence to support the utility of GRSs to identify people with diabetes who are at high risk of developing DKD. Further prospective research is needed to strengthen the evidence for the utility of GRSs to predict DKD in clinical practice. Once a robust GRS for DKD has been established, future research can focus on its integration into clinical workflow and its impact on clinical outcomes.

## Data Availability

Original data generated and analyzed during this study are included in this published articles or in the data repositories listed in the References. PROSPERO URL: https://www.crd.york.ac.uk/prospero/display_record.php?RecordID=402057. PROSPERO ID: CRD42023402057.

## References

[1] Alicic RZ, Rooney MT, Tuttle KR. Diabetic kidney disease: challenges, progress, and possibilities. Clin J Am Soc Nephrol. 2017;12(12):2032‐2045. 28522654 10.2215/CJN.11491116 PMC5718284

[2] Hussain S, Chand Jamali M, Habib A, Hussain MS, Akhtar M, Najmi AK. Diabetic kidney disease: an overview of prevalence, risk factors, and biomarkers. Clin Epidemiol Glob Health. 2021;9:2‐6.

[3] White S, Chadban S. Diabetic kidney disease in Australia: current burden and future projections. Nephrology. 2014;19(8):450‐458. 24888506 10.1111/nep.12281

[4] Adler AI, Stevens RJ, Manley SE, Bilous RW, Cull CA, Holman RR. Development and progression of nephropathy in type 2 diabetes: the United Kingdom prospective diabetes study (UKPDS 64). Kidney Int. 2003;63(1):225‐232. 12472787 10.1046/j.1523-1755.2003.00712.x

[5] KDGIO . KDIGO 2012 clinical practice guideline for the evaluation and management of chronic kidney disease. Kid Int Supp. 2013;3(1):19‐62. 10.1038/ki.2013.243 23989362

[6] Tuttle KR, Bakris GL, Bilous RW, et al Diabetic kidney disease: a report from an ADA consensus conference. Diabetes Care. 2014;37(10):2864‐2883. 25249672 10.2337/dc14-1296 PMC4170131

[7] Liu L, Kiryluk K. Genome-wide polygenic risk predictors for kidney disease. Nat Rev Nephrol. 2018;14(12):723‐724. 30279535 10.1038/s41581-018-0067-6 PMC8404375

[8] CASP Checklists Oxford, UK: critical appraisal skills programme; 2022. Available from: https://casp-uk.net/casp-tools-checklists/.

[9] Ali AS . Methodology quality assesment. 2023: 1‐3.

[10] Todd JN, Dahlstrom EH, Salem RM, et al Genetic evidence for a causal role of obesity in diabetic kidney disease. Diabetes. 2015;64(12):4238‐4246. 26307587 10.2337/db15-0254 PMC4657582

[11] van Zuydam NR, Ahlqvist E, Sandholm N, Deshmukh H, Rayner NW, Abdalla M. A genome-wide association study of diabetic kidney disease in subjects with type 2 diabetes. Diabetes. 2018;67(7):1414‐1447. 29703844 10.2337/db17-0914 PMC6014557

[12] Liao L-N, Li T-C, Li C-I, et al Genetic risk score for risk prediction of diabetic nephropathy in Han Chinese type 2 diabetes patients. Sci Rep. 2019;9(1):19897. 31882689 10.1038/s41598-019-56400-3 PMC6934611

[13] Barbieux P, Gyorgy B, Gand E, et al No prognostic role of a GWAS-derived genetic risk score in renal outcomes for patients from French cohorts with type 1 and type 2 diabetes. Diabetes Metab. 2019;45(5):494‐497. 29540294 10.1016/j.diabet.2018.01.016

[14] Zusi C, Trombetta M, Bonetti S, et al A renal genetic risk score (GRS) is associated with kidney dysfunction in people with type 2 diabetes. Diabetes Res Clin Pract. 2018;144:137‐143. 30153470 10.1016/j.diabres.2018.08.013

[15] Vujkovic M, Keaton JM, Lynch JA, et al Discovery of 318 new risk loci for type 2 diabetes and related vascular outcomes among 1.4 million participants in a multi-ancestry meta-analysis. Nat Genet. 2020;52(7):680‐691. 32541925 10.1038/s41588-020-0637-y PMC7343592

[16] Tremblay J, Haloui M, Attaoua R, et al Polygenic risk scores predict diabetes complications and their response to intensive blood pressure and glucose control. Diabetologia. 2021;64(9):2012‐2025. 34226943 10.1007/s00125-021-05491-7 PMC8382653

[17] Hsieh A-R, Huang Y-C, Yang Y-F, et al Lack of association of genetic variants for diabetic retinopathy in Taiwanese patients with diabetic nephropathy. BMJ Open Diabetes Res Care. 2020;8(1):e000727. 10.1136/bmjdrc-2019-000727 PMC7039583 31958309

[18] Ahlqvist E, Storm P, Käräjämäk A, Martinell M, Dorkhan M. Novel subgroups of adult-onset diabetes and their association with outcomes: a data-driven cluster analysis of six variables. Lancet Diabetes Endocrinol. 2018;6(5):361‐369. 29503172 10.1016/S2213-8587(18)30051-2

[19] Wang J, Liu J-J, Gurung RL, et al Clinical variable-based cluster analysis identifies novel subgroups with a distinct genetic signature, lipidomic pattern and cardio-renal risks in Asian patients with recent-onset type 2 diabetes. Diabetologia. 2022;65(12): 2146‐2156. 35763031 10.1007/s00125-022-05741-2 PMC9630229

[20] Wang Y, Luk AOY, Ma RCW, et al Predictive role of multilocus genetic polymorphisms in cardiovascular disease and inflammation-related genes on chronic kidney disease in type 2 diabetes–an 8-year prospective cohort analysis of 1163 patients. Nephrol Dialysis Transplant. 2012;27(1):190‐196. 10.1093/ndt/gfr343 21765051

[21] Slieker RC, Donnelly LA, Fitipaldi H, et al Distinct molecular signatures of clinical clusters in people with type 2 diabetes: an IMI-RHAPSODY study. Diabetes. 2021;70(11):2683‐2693. 34376475 10.2337/db20-1281 PMC8564413

[22] Xu M, Bi Y, Huang Y, et al Type 2 diabetes, diabetes genetic score and risk of decreased renal function and albuminuria: a Mendelian randomization study. eBioMedicine. 2016;6:162‐170. 27211558 10.1016/j.ebiom.2016.02.032 PMC4856750

[23] Rattanatham R, Settasatian N, Komanasin N, et al Association of combined TCF7L2 and KCNQ1 gene polymorphisms with diabetic micro- and macrovascular complications in type 2 diabetes Mellitus. Diabetes Metab J. 2021;45(4):578‐593. 33752320 10.4093/dmj.2020.0101 PMC8369220

[24] Gurung RL, Yiamunaa M, Liu J-J, et al Genetic risk score for plasma uric acid levels is associated with early rapid kidney function decline in type 2 diabetes. J Clin Endocrinol Metab. 2022;107(7):e2792‐e2800. 35363857 10.1210/clinem/dgac192

[25] Bakris GL, Agarwal R, Anker SD, et al Effect of finerenone on chronic kidney disease outcomes in type 2 diabetes. N Engl J Med. 2020;383(23):2219‐2229. 33264825 10.1056/NEJMoa2025845

[26] Brenner BM, Cooper ME, de Zeeuw D, et al Effects of losartan on renal and cardiovascular outcomes in patients with type 2 diabetes and nephropathy. N Engl J Med. 2001;345(12):861‐869. 11565518 10.1056/NEJMoa011161

[27] Brown E, Heerspink HJL, Cuthbertson DJ, Wilding JPH. SGLT2 inhibitors and GLP-1 receptor agonists: established and emerging indications. Lancet. 2021;398(10296):262‐276. 34216571 10.1016/S0140-6736(21)00536-5

[28] Zhang L, Long J, Jiang W, et al Trends in chronic kidney disease in China. New Engl J Med. 2016;375(9):905‐906. 27579659 10.1056/NEJMc1602469

[29] Chatterjee N, Shi J, García-Closas M. Developing and evaluating polygenic risk prediction models for stratified disease prevention. Nat Rev Genet. 2016;17(7):392‐406. 27140283 10.1038/nrg.2016.27 PMC6021129

[30] Mavaddat N, Pharoah PD, Michailidou K, et al Prediction of breast cancer risk based on profiling with common genetic variants. J Natl Cancer Inst. 2015;107(5):djv036. 25855707 10.1093/jnci/djv036 PMC4754625

[31] Khera AV, Chaffin M, Aragam KG, et al Genome-wide polygenic scores for common diseases identify individuals with risk equivalent to monogenic mutations. Nat Genet. 2018;50(9):1219‐1224. 30104762 10.1038/s41588-018-0183-z PMC6128408

[32] Lang F, Rehwald W. Potassium channels in renal epithelial transport regulation. Physiol Rev. 1992;72(1):1‐32. 1731368 10.1152/physrev.1992.72.1.1

[33] Yasuda K, Miyake K, Horikawa Y, et al Variants in KCNQ1 are associated with susceptibility to type 2 diabetes mellitus. Nat Genet. 2008;40(9):1092‐1097. 18711367 10.1038/ng.207

[34] Ohshige T, Tanaka Y, Araki S, et al A single nucleotide polymorphism in KCNQ1 is associated with susceptibility to diabetic nephropathy in Japanese subjects with type 2 diabetes. Diabetes Care. 2010;33(4):842‐846. 20056949 10.2337/dc09-1933 PMC2845039

[35] Lim XL, Nurbaya S, Salim A, et al KCNQ1 SNPS and susceptibility to diabetic nephropathy in East Asians with type 2 diabetes. Diabetologia. 2012;55(9):2402‐2406. 22696034 10.1007/s00125-012-2602-5

